# Sustainable Carbon Nanomaterials from Biomass Precursors: Green Synthesis Strategies and Environmental Applications

**DOI:** 10.3390/nano16010075

**Published:** 2026-01-05

**Authors:** Ernesto Almaraz-Vega, Aislinn Itzel Morales-Vargas, Guillermo Gómez Delgado, Laura Castellanos-Arteaga, Ofelia Iñiguez Gómez, Claudia Cecilia Flores Salcedo

**Affiliations:** 1 Chemical Engineering Department, University of Guadalajara (CUCEI), Blvd. Marcelino García Barragán #1421, esq. Calzada Olímpica, Guadalajara 44430, Jalisco, Mexico; ernesto.almaraz5309@academicos.udg.mx (E.A.-V.); aislinn.morales@alumnos.udg.mx (A.I.M.-V.); 2 Natural and Health Sciences, Tepatitlan Regional Preparatory School, High School Education System, University of Guadalajara, Tepatitlan de Morelos 47600, Jalisco, Mexico; claudia.flores1709@academicos.udg.mx; 3 Natural and Health Sciences, Tepatitlan Regional Preparatory School, Acatic Module, High School Education System, University of Guadalajara, Acatic 45470, Jalisco, Mexico; laura.castellanos@academicos.udg.mx; 4 Animal Sciences and Engineering Department, University of Guadalajara (CUALTOS), Carretera a Yahualica km 7.5, Tepatitlán de Morelos 47600, Jalisco, Mexico; ofelia.iniguez@academicos.udg.mx

**Keywords:** biomass-derived carbon nanomaterials, green synthesis, adsorption, photocatalysis, environmental remediation

## Abstract

Environmental pollution caused by industrialization and population growth has intensified the demand for sustainable materials capable of mitigating contaminants effectively. In this context, the green synthesis of carbon-based nanomaterials derived from biomass has gained significant attention as an eco-friendly and renewable approach that reduces dependence on fossil resources. These nanomaterials exhibit outstanding physicochemical characteristics, including high surface area, tunable porosity, abundant functional groups, and excellent stability, which enhance their performance in environmental remediation. Specifically, biomass-derived carbon nanomaterials have demonstrated remarkable efficiency as adsorbents for the removal of heavy metals and organic pollutants, as well as photocatalysts for the degradation of toxic compounds under visible light irradiation. The physicochemical properties of the resulting materials are strongly influenced by the type and pretreatment of the biomass, along with synthesis parameters such as pyrolysis temperature, activation process, and heteroatom doping. This review highlights recent advances in the synthesis, characterization, and environmental applications of biomass-derived carbon nanomaterials, emphasizing their potential as cost-effective, scalable, and sustainable solutions for wastewater treatment and pollutant degradation in both aquatic and atmospheric systems.

## 1. Introduction

### 1.1. Background and Motivation

The exponential growth of the global population and the accelerated pace of industrial development have generated an increasing demand for energy resources, raw materials, and technologies for pollutant treatment. As a result, pressure on ecosystems has intensified, exacerbating issues such as climate change, depletion of natural resources, and the pollution of water, air, and soil [1]. Under these circumstances, the search for sustainable solutions has become a scientific and technological priority, not only to mitigate environmental impacts but also to ensure energy security and access to clean technologies.

Carbon nanomaterials (CNMs) have emerged as a class of materials with exceptional structural, electrical, thermal, and chemical properties, making them highly attractive for environmental applications, including pollutant adsorption, catalysis, water treatment, and sensing [2,3]. Their high specific surface area, versatile surface functionalization, and chemical stability position them as key components in emerging technologies that support the transition toward a circular economy.

However, conventional methods for synthesizing these nanomaterials (such as chemical vapor deposition (CVD), laser ablation, and the pyrolysis of fossil-based precursors) often require high temperatures, metallic catalysts, and petroleum-derived toxic substances [4,5,6]. These factors contradict the principles of green chemistry and limit industrial scalability in terms of cost, safety, and sustainability [7]. Consequently, recent years have seen growing research interest in alternative synthesis routes that utilize renewable carbon sources, such as agro-industrial waste and lignocellulosic biomass, under more environmentally friendly conditions.

In parallel, the development of technologies that integrate concepts of circular economy, waste valorization, and green synthesis has gained strategic importance in both developed and emerging economies [8]. Such strategies not only enable cleaner production of nanomaterials but also promote the utilization of residues that would otherwise represent an environmental burden.

Accordingly, a clear scientific need exists to understand, integrate, and optimize green methodologies for the production of carbon nanomaterials, particularly those employing biomass as a raw material. This review addresses this need by contributing to the design of functional materials through sustainable routes compatible with the environmental challenges of the twenty-first century.

### 1.2. Biomass as Renewable Feedstock

The choice of precursor is a critical factor in the synthesis of carbon nanomaterials. Traditionally, many of these materials have been produced from petroleum-derived precursors, such as methane, acetylene, benzene, and other highly purified organic substances [9,10,11]. Although these compounds allow for precise control over certain characteristics of the final product, their use involves several drawbacks, including dependence on non-renewable resources, high production costs, a significant carbon footprint, toxicity risks, and intensive energy consumption during transformation processes.

In contrast, lignocellulosic biomass and other organic residues constitute a sustainable, cost-effective, and environmentally favorable alternative for the production of carbon nanomaterials. Biomass is defined as any organic material of plant or animal origin that can be converted into energy or functional materials. Representative examples include agricultural residues (such as rice husk, sugarcane bagasse, leaves, and stems), forestry residues, algae, and by-products from the food industry [12].

One of the main advantages of biomass over fossil precursors lies in its renewability. Unlike petroleum, which requires millions of years to form, biomass can regenerate over relatively short timescales, even within annual cycles. In addition, its abundance and global availability contribute to cost reduction and promote circular economic models in rural communities and regions with high agricultural activity.

Another important advantage is the lower ecological footprint associated with biomass. Its transformation into nanomaterials can be achieved through low-temperature processes, such as slow pyrolysis or hydrothermal carbonization, resulting in energy consumption reductions of up to 30% compared with conventional chemical synthesis methods [13]. Furthermore, the use of waste materials prevents competition with food crops and alleviates pressure on landfills and incineration facilities, thereby contributing to the integrated management of municipal solid waste.

From a structural perspective, biomass contains high levels of carbon (in the form of cellulose, hemicellulose, and lignin) as well as heteroatoms such as oxygen, nitrogen, sulfur, and phosphorus. This intrinsic composition enables the production of functionalized or doped nanomaterials without the need for toxic or costly additives, which is particularly advantageous for environmental applications, including heavy metal adsorption, degradation of organic pollutants, and wastewater remediation [13,14,15].

Moreover, the use of biomass supports the development of more economical and scalable processes, which is essential for the transition toward green and industrially viable nanotechnology. The ability to synthesize functional materials from waste without intensive pretreatment represents a significant step toward the sustainability of emerging technologies [16,17,18].

Despite the advantages associated with biomass-derived precursors, it is important to emphasize that the use of renewable feedstocks alone does not inherently guarantee an environmentally benign synthesis process. The overall environmental impact of carbon nanomaterial production is strongly influenced by additional factors, including energy demand, the use of chemical activation agents, reaction conditions, and post-treatment steps such as purification or surface functionalization. In some cases, these stages may involve high temperatures, corrosive chemicals, or intensive water and energy consumption, which can offset the sustainability benefits associated with the renewable origin of the precursor. Accordingly, in the context of this review, the term “green” is not used as a blanket descriptor of biomass-based routes, but rather as a conditional attribute that must be evaluated across the entire life cycle of the material. Similarly, the “advances” discussed herein should not be interpreted solely as increases in material performance or synthesis efficiency, but as progress toward more rational, transparent, and sustainability-aware design strategies that explicitly balance functional properties with environmental trade-offs. This distinction underscores the need to differentiate between renewable sourcing, technological improvement, and genuine environmental performance when assessing the true significance of recent developments in biomass-derived carbon nanomaterials.

### 1.3. Scope and Structure of the Review

Several review articles have addressed the synthesis of carbon nanomaterials from biomass, primarily focusing on summarizing available synthesis routes, classifying precursor sources, and enumerating potential environmental applications. These studies provide valuable descriptive overviews of processing techniques and reported functionalities; however, they often treat synthesis methods, material properties, and applications as largely independent topics. As a result, direct comparisons between synthesis conditions, resulting physicochemical properties, and actual application performance remain limited. In addition, many existing reviews adopt the term “green” in a broad or qualitative manner, frequently equating sustainability with the renewable origin of the precursor, without defining operational criteria related to energy demand, chemical inputs, or overall environmental impact. Furthermore, systematic contrasts between biomass-derived carbon nanomaterials and those produced from fossil-based precursors (in terms of performance, efficiency, and environmental trade-offs) are rarely explored in depth. In this context, the present review aims to provide a structured and critical framework that explicitly links synthesis strategies to material properties and, ultimately, to application performance. By integrating synthesis conditions, structural and surface characteristics, and functional outcomes, this work enables a comparative evaluation of biomass-derived carbon nanomaterials across different environmental applications. Moreover, this review identifies current limitations, knowledge gaps, and unresolved challenges related to scalability, sustainability, and performance benchmarking, thereby highlighting key research directions necessary for advancing truly green and competitive carbon nanomaterial technologies. The objective of this review is to provide a comprehensive overview of recent advances in the green synthesis of carbon nanomaterials derived from biomass, with particular emphasis on their environmental applications. To this end, the discussion addresses the classification and characteristics of different biomass sources, as well as the various synthesis strategies employed, including both conventional and advanced approaches.

Subsequently, the most relevant types of nanomaterials obtained are examined, including biochar, carbon dots, graphene, and carbon nanotubes, together with the functionalization techniques used to optimize their performance in specific applications. Key environmental applications (such as pollutant adsorption, photocatalysis, and electrochemical water treatment) are also discussed.

This review aims to critically analyze biomass-derived carbon nanomaterials by systematically correlating synthesis parameters, physicochemical properties, and environmental application performance, while assessing their true sustainability potential. Finally, the main challenges in this field are considered, including standardization, scalability, and environmental impact, concluding with a reflection on the importance of advancing toward a circular and sustainable economy.

## 2. Review Methodology

### 2.1. Literature Search Strategy

A systematic literature search was conducted to identify relevant studies on biomass-derived carbon nanomaterials and their environmental applications. The primary databases consulted were Web of Science and Scopus, which were selected due to their broad coverage of peer-reviewed scientific literature in materials science and environmental engineering. Google Scholar was used only as a complementary source to identify additional relevant publications not indexed in the primary databases.

The search covered publications from 2008 to 2025, reflecting the period of most rapid development in green synthesis strategies for carbon nanomaterials. Search terms were grouped conceptually and combined using Boolean operators, including keywords related to (i) biomass and renewable carbon sources, (ii) carbon nanomaterials (e.g., biochar, carbon dots, graphene, carbon nanotubes), (iii) green or low-impact synthesis methods, and (iv) environmental applications such as adsorption, photocatalysis, and water treatment.

### 2.2. Inclusion and Exclusion Criteria

To ensure transparency, consistency, and reproducibility, explicit inclusion and exclusion criteria were applied during the selection of publications.

Inclusion criteria comprised studies reporting the synthesis of carbon nanomaterials derived from biomass or organic waste, employing synthesis routes described as green, sustainable, or low impact. Selected studies were required to include clear physicochemical characterization of the materials (e.g., surface area, porosity, structural features, or surface chemistry) and to report quantitative performance data in at least one environmental application, such as pollutant adsorption, photocatalysis, or electrochemical water treatment.

Exclusion criteria included studies focused on commercial or fossil-derived carbon materials without clear precursor traceability, reports lacking adequate material characterization, and works that were purely theoretical, conceptual, or descriptive without experimental validation. Publications addressing carbon materials not derived from biomass or renewable sources were also excluded.

## 3. Biomass Precursors for Carbon Nanomaterials

### 3.1. Classification of Biomass Sources

Biomass represents an abundant, renewable, and cost-effective source of raw material for the production of carbon nanomaterials. Its complex organic composition, rich in carbon, makes it a viable and sustainable alternative to petroleum-derived precursors. Biomass used in the synthesis of nanomaterials can be classified into different categories according to their origin and physicochemical characteristics.

#### 3.1.1. Agricultural Residues

**Figure 1:**
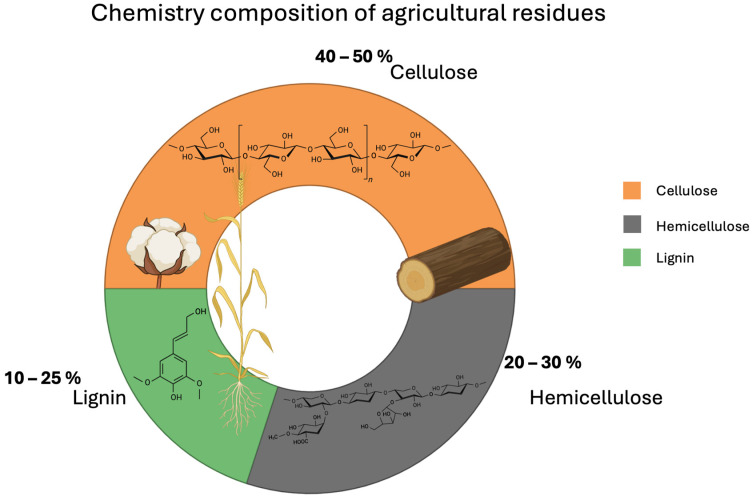
Chemical composition of these agricultural residues.

Agricultural lignocellulosic biomass represents an abundant and renewable source of raw material for the synthesis of carbon nanomaterials due to its high carbon content and global availability. This type of biomass is primarily composed of three structural components: cellulose, hemicellulose, and lignin. In general terms, as shown in Figure 1, the chemical composition of these agricultural residues ranges from 40 to 50% cellulose, 20–30% hemicellulose, and 10–25% lignin, depending on the crop type and processing conditions [19]. For example, corn stover has been reported to contain 37.4% cellulose, 21.1% xylan (the main hemicellulose), and 18% lignin [20]. Similarly, sugarcane bagasse, a common by-product in tropical regions, contains between 26 and 50% cellulose, 24–34% hemicellulose, and 10–26% lignin [21]. These proportions make these residues suitable for valorization applications, particularly in the production of materials with a high surface-to-volume ratio, such as carbon nanomaterials.

Agricultural residues can include straw, husks, stems, and other by-products generated after harvests. This type of biomass is particularly attractive due to its seasonal availability and low cost. In addition, its high cellulose and hemicellulose content favors the formation of carbonaceous structures during pyrolysis [22,23].

Beyond availability, the use of agricultural residues offers significant environmental advantages. Utilizing these wastes contributes to a circular economy by reducing the amount of agricultural waste that is commonly burned or improperly disposed of. Their conversion into advanced materials such as carbon nanomaterials can decrease reliance on fossil resources, reduce greenhouse gas emissions, and promote more sustainable processes [24,25,26].

The most commonly employed conversion routes for transforming biomass into carbon nanomaterials include controlled pyrolysis, hydrothermal treatment, and high-temperature carbonization [27,28]. These techniques allow modification of the structure and morphology of the resulting carbon, influencing surface properties and the material’s ability to interact with contaminants or catalysts [27]. The choice of method depends on the type of biomass, process design, and desired properties of the final product.

Carbon nanomaterials derived from biomass have demonstrated outstanding performance in various environmental applications, including heavy metal adsorption [28], removal of dyes [29] and emerging contaminants [30], and use as supports in heterogeneous catalysis [28,31]. Furthermore, they have been applied in energy storage technologies such as supercapacitors and batteries, showing performance comparable to conventional synthetic materials [32,33].

Nevertheless, there are limitations and challenges that must be addressed. Variability in biomass composition, difficulty in controlling nanoscale structure, and the need for efficient purification processes are some of the main obstacles. In addition, industrial scalability and the lack of standardization in the final products may hinder commercial implementation.

#### 3.1.2. Lignocellulosic Waste

Lignocellulosic residues constitute one of the most abundant renewable sources of organic carbon on the planet, with an estimated annual production of up to 200 billion tons [34]. This type of residue encompasses a wide variety of plant-derived materials, including agricultural by-products (such as straw, bagasse, or husks), forestry residues (such as sawdust or bark), woody crops, and even a significant fraction of municipal solid waste, which can contain up to 60% lignocellulose on a dry weight basis [35].

Although often grouped with agricultural residues, lignocellulosic wastes exhibit particular structural characteristics. While agricultural residues may include a broader range of easily biodegradable organic materials (such as fruit and vegetable scraps or manure), lignocellulosic residues are distinguished by their complex and rigid structure, primarily composed of cellulose, hemicellulose, and lignin. This configuration provides high stability and resistance to microbial degradation, making direct conversion into products such as biogas, bioethanol, or nanomaterials challenging without a prior pretreatment step [36,37,38].

Their high energy content, in relation to biomass yield and available cultivable area [39,40], has motivated the development of valorization technologies. However, their structural recalcitrance requires hydrolysis or deconstruction processes to break the lignin-hemicellulose network that protects cellulose microfibrils. The efficiency of these processes depends on biomass type, cellulose crystallinity, chemical composition, and treatments applied to the raw material, such as drying or heating [41]. Various physicochemical, biological, and thermochemical pretreatment methods are currently being investigated to improve digestibility and facilitate the conversion of these residues into high-value products, including biofuels, biopolymers, or carbon nanomaterials.

#### 3.1.3. Vegetable Oils (Including Waste Oils)

Vegetable oils, both virgin and waste-derived, have emerged as sustainable and highly effective precursors for carbon nanomaterial synthesis due to their high carbon content and low levels of inorganic impurities. These materials predominantly contain triglycerides composed of glycerol and fatty acids, making them a rich carbon source for pyrolysis or chemical vapor deposition (CVD) processes [42,43]. Unlike other lignocellulosic residues, vegetable oils do not require complex pretreatment or prior depolymerization steps, which makes them attractive from both technical and economic perspectives.

Several studies have reported successful production of nanomaterials such as carbon nanofibers (CNFs), carbon nanotubes (CNTs), and graphene from oils including soybean, safflower, sunflower, rapeseed, and waste cooking oil [44,45]. For instance, catalytic CVD techniques have demonstrated that palm oil is an effective precursor for CNTs with high crystallinity and purity [46]. Moreover, these oils allow precise control over nanomaterial morphology by adjusting parameters such as reaction temperature, catalyst type, and carrier gas flow.

Nevertheless, challenges associated with oil use remain, particularly regarding variability in chemical composition, which can affect reproducibility of the synthesized nanomaterial properties. Furthermore, industrial-scale technologies for their utilization are still limited, highlighting the need for further research focused on process standardization and carbon yield improvement.

#### 3.1.4. Algae and Aquatic Biomass

Algae and aquatic biomass have emerged as versatile and sustainable precursors for carbon nanomaterial synthesis due to their availability, rapid growth rates, and high biomass productivity per unit area [47,48]. Unlike other lignocellulosic sources or vegetable oils, algae do not compete with food crops or require large areas of agricultural land, positioning them as a key raw material within the circular bioeconomy and green chemistry frameworks.

Microalgae, macroalgae, and certain cyanobacteria contain organic compounds such as lipids, carbohydrates, proteins, and pigments, which act as reducing and stabilizing agents during green nanomaterial synthesis [49]. These metabolites facilitate the production of metal nanoparticles (e.g., Au, Ag), metal oxides (e.g., ZnO) [50], and carbon-based materials such as carbon dots and graphene-like structures through processes such as hydrothermal carbonization [51].

However, the effectiveness of these nanomaterials is strongly influenced by the algal species used, its chemical composition, and the cultivation environmental conditions. This poses challenges for process standardization and reproducibility. Moreover, synthesis routes must be optimized to control size, morphology, and purity, which are critical parameters for specific applications [52,53].

Overall, algae and other forms of aquatic biomass offer a promising route for sustainable production of functional nanomaterials with high added value and low environmental impact. Industrial integration will depend on the development of efficient, scalable, and reproducible production techniques capable of meeting the required standards for each application.

#### 3.1.5. Food Industry and Urban Organic Waste

Waste generated by the food industry and urban organic waste represent an abundant, economical, and renewable source for green synthesis of nanomaterials. These wastes include wheat bran, sugarcane bagasse char, food waste, orange peels, onion residues, wheat straw, and bamboo residues, among many others [54], which possess a high content of carbon-rich organic compounds and functional biomolecules capable of acting as precursors and reducing agents in sustainable synthesis processes.

Kang and colleagues have shown that biomass residues represent a promising alternative for the production of carbon dots, offering key advantages such as abundance, broad availability, and ecological compatibility [54]. Additionally, these residues constitute a renewable and low-cost source of various raw materials (including cellulose, hemicellulose, lignin, carbohydrates, and proteins), making them ideal resources for the development of sustainable materials with potential applications across multiple technological fields.

Table 1 summarizes some of the most relevant advances in recent years regarding the synthesis of various carbon nanostructures derived from biomass wastes. This table complements the one reported by Santosh and colleagues [27], which provides similar information and contributes additional context on recent developments in the field. The table shows that the wide variety of raw materials used (such as fruit peels, leaves, agricultural residues, and food waste) not only demonstrates the significant potential for valorization of these resources but also reflects the versatility of the conversion techniques employed. These developments open new opportunities for the sustainable production of nanomaterials with applications ranging from metal and contaminant detection to catalysis, bioimaging, and chemical sensing, contributing to a more ecological and efficient approach to the design of functional materials.

### 3.2. Chemical Composition and Characteristics

The chemical composition of precursor biomasses plays a fundamental role in the structure, yield, and properties of carbon nanomaterials obtained via green synthesis. Among the main constituents, cellulose, hemicellulose, and lignin possess distinct molecular structures and physicochemical characteristics that directly influence their behavior during carbonization and their potential for functionalization.

#### Cellulose, Hemicellulose, and Lignin

Cellulose is a complex carbohydrate found in plant cell walls, providing rigidity and structural strength. It consists of 7000–15,000 [78] repetitive units of β-D-glucose linked by β-1,4-glycosidic bonds [79]. Its linear arrangement results in a semicrystalline structure composed of long chains of D-glucopyranose units regularly joined.

**Figure 2:**
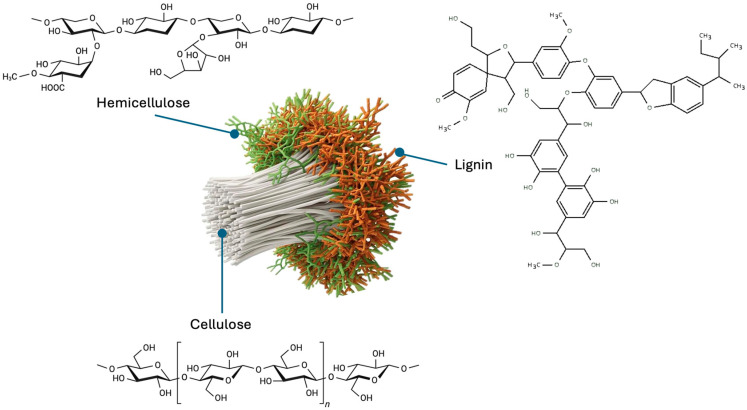
The organization of three components in lignocellulosic biomass.

On the other hand, hemicellulose is a low-degree polymerization copolymer (*n* = 500–3000) [78], a heterogeneous and amorphous polysaccharide composed mainly of pentoses, hexoses, and uronic acids. Its structure is formed by β-(1→4) linkages with equatorial configuration, to which various side chains are attached. This group includes xylans, mannans, glucomannans, xyloglucans, and in certain cases such as *Poales*, β-(1→3,1→4)-glucans [80]. The composition and abundance of hemicelluloses vary considerably among plant species and cell types, resulting in differences in their chemical and functional properties. Their lower thermal stability and branched conformation promote faster decomposition during thermochemical processes [81], facilitating the release of volatiles and the formation of porosity in carbon nanomaterials. Figure 2 illustrates the structure of cellulose, hemicellulose, and lignin.

In contrast to hemicellulose, lignin represents a structural component of aromatic nature that provides rigidity, mechanical strength, and impermeability to the cell wall. This complex polymer is formed by randomly cross-linked networks of hydroxylated and methoxylated phenylpropane units [82], which confer high structural heterogeneity. Due to its aromatic character and the abundance of ether and carbon–carbon bonds, lignin is more resistant to thermal degradation [81] than the polysaccharides in the cell wall, making its natural decomposition difficult but rendering it an ideal source for producing carbonaceous materials with high chemical stability.

## 4. Types and Properties of Biomass-Derived Carbon Nanomaterials

### 4.1. Biochar and Activated Carbon

Biochar is a porous carbonaceous material obtained from the carbonization of biomass (via fast or slow pyrolysis or hydrothermal treatments) [83]. The structure of biochar is primarily microporous, although the ratio of micropores to mesopores depends on the processing conditions and the type of biomass used, with relatively low specific surface areas (typically between 1 and 400 m^2^/g) [84,85], unless it is subsequently activated. For example, biochar from corn starch can exhibit surface areas of only ~1–7 m^2^/g, indicating limited porosity [86]. Chemical or physical activation (usually with KOH, CO_2_, or steam) drastically increases the surface area, reaching values from 500 to over 2000 m^2^/g [87,88,89]. These materials contain numerous residual oxygenated functional groups (–OH, –COOH) on their surface [90,91,92].

Activated carbon (AC) is obtained by treating biochar with oxidizing agents (steam, CO_2_, or alkalis such as KOH), producing a highly developed porous network. These processes, known as physical (steam, CO_2_) or chemical activation (KOH, ZnCl_2_, K_2_CO_3_), generate a highly porous structure and significantly increase the surface area of the resulting material [93,94,95].

In addition to increasing surface area, activation significantly modifies the pore size distribution in activated carbon. Generally, these materials feature a high proportion of micropores (<2 nm) responsible for high adsorption capacities for gases and organic contaminants, often accompanied by mesopores (2–50 nm) that facilitate diffusion of larger molecules and improve adsorption kinetics. The presence of macropores (>50 nm), although less abundant, acts as transport pathways to deeper regions of the structure. The relative proportion of micro-, meso-, and macropores depends on the precursor used and the activation method: while physical activation with steam or CO_2_ typically produces carbons with surface areas in the range of 500–1200 m^2^/g [96] dominated by micropores, chemical activation with KOH can reach values close to 2000 m^2^/g [97], with a hierarchical pore distribution including a significant fraction of mesopores. This combination of high specific surface area and diversified porous structure substantially expands the applications of activated carbon in the adsorption of large contaminants, energy storage (supercapacitors, batteries), and heterogeneous catalysis [98].

### 4.2. Carbon Dots

Carbon dots (CDs) are zero-dimensional carbon nanomaterials, typically smaller than 10 nm, notable for their high water solubility, low toxicity, good biocompatibility, strong photoluminescent stability, and ease of surface functionalization [99,100].

Biomass-derived carbon dots have shown great potential in various fields due to their optical properties and biocompatibility. In bioimaging and biosensors, their low toxicity, luminescent stability, and aqueous solubility allow their use as safe fluorescent probes for cellular visualization and biomolecule detection [100,101]. They have also been applied to detect environmental contaminants, such as heavy metal ions, showing high sensitivity and submicromolar detection limits, for example, in the detection of Fe(III) in water [102]. In the energy sector, they have been used as active components in supercapacitors and optoelectronic devices (OLEDs), leveraging their high conductivity and emission properties. Additionally, their ability to emit multicolor fluorescence makes them useful in security and anti-counterfeiting applications, acting as “invisible inks” for marking and protecting documents or products [103].

### 4.3. Graphene and Graphene Oxide

Graphene is a two-dimensional monolayer of sp^2^-hybridized carbon atoms arranged in a hexagonal honeycomb lattice. Each carbon atom contributes a delocalized π orbital, which provides exceptional electronic mobility and allows ballistic electron transport at the nanoscale [104]. These structural characteristics explain its extremely high electrical conductivity, reported in the range of 10^5^–10^6^ S/m [105], as well as its outstanding mechanical strength and elastic modulus, which far exceed those of most conventional materials [106].

Graphene oxide (GO), derived from graphene, consists of sheets functionalized with oxygen-containing groups such as epoxides, hydroxyls, and carboxyls [107]. The presence of these functionalities disrupts the conjugated π network, significantly reducing electrical conductivity [108]. However, these same oxygen groups provide GO with high hydrophilicity and dispersibility in aqueous media [109], advantageous for applications in filtration membranes, sensors, and nanocomposites.

**Figure 3:**
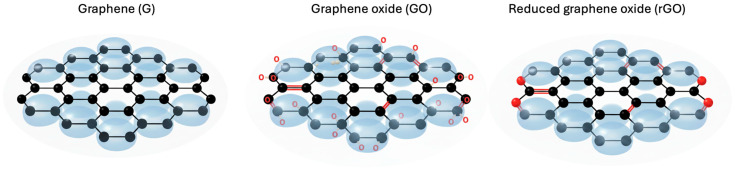
Structures of graphene (G), graphene oxide (GO), and reduced graphene oxide (rGO).

When GO undergoes reduction processes (chemical, thermal, or electrochemical), the resulting material is reduced graphene oxide (rGO) [107]. This material partially recovers electrical conductivity due to the removal of oxygenated groups, although structural defects in the sheets result in electronic properties still inferior to pristine graphene. Nevertheless, rGO retains advantages such as processability and chemical affinity, making it a versatile material for applications in energy, catalysis, and electronic devices. Figure 3 illustrates the structural differences among graphene, graphene oxide, and reduced graphene oxide, highlighting variations in their atomic organization and the presence of functional groups.

A notable example of biomass-derived rGO is the in situ synthesis of α-Fe_2_O_3_/ZnO/rGO heterojunctions with coral-like morphology using basil seeds as a sustainable precursor. This material was successfully applied for the photodegradation of oxytetracycline (OTC), a common antibiotic and emerging water contaminant. The photocatalyst achieved 98% degradation in 90 min under visible light in a helical-piston flow photoreactor (HPFPR), reaching a reaction rate 2–3 times higher than batch reactors [110]. This example demonstrates how biomass-derived rGO can be integrated into advanced photocatalytic systems, offering sustainable and scalable solutions for wastewater remediation of antibiotics, with direct implications for environmental protection and public health.

Additionally, agricultural residues have also been used as precursors for rGO synthesis with biomedical applications, as demonstrated by *Setaria italica* biowaste reduced with *Prosopis juliflora* phytochemicals to develop a biocompatible hydrogel with antioxidant, antibacterial, and anti-inflammatory properties, proposed as a promising material for wound dressings [111].

In summary, pristine graphene, GO, and rGO represent a family of nanomaterials with complementary structural and electronic properties. While graphene excels in conductivity and mechanical strength, GO and rGO offer chemical functionality and improved compatibility with polymeric and inorganic matrices, expanding their potential in fields such as environmental remediation, energy storage, and advanced sensor development.

### 4.4. Carbon Nanotubes and Nanofibers

Carbon nanotubes (CNTs) are hollow cylindrical structures formed by rolling graphene sheets, with length-to-diameter ratios exceeding 1,000,000 [112]. Depending on their configuration, they can be classified as single-walled carbon nanotubes (SWCNTs) or multi-walled carbon nanotubes (MWCNTs) [113], the latter consisting of several concentric layers. SWCNT diameters typically range from 0.5 to 5 nm, whereas MWCNTs have diameters of several tens of nanometers and lengths that can reach the micrometer scale [114].

One of their most remarkable features is their mechanical properties. Theoretically, a single-walled nanotube can withstand tensile stresses of up to 100–200 GPa, with a Young’s modulus close to 1 TPa [115]. Experimentally, values reach tens of GPa, which is still far superior to conventional materials. For example, typical steel exhibits a tensile strength around 0.5 GPa, so even MWCNTs, with their more complex concentric structures, achieve strengths on the order of 10–50 GPa, making them exceptional reinforcements for advanced structural applications.

Regarding their electronic properties, CNTs can behave as metallic conductors or semiconductors depending on their chirality [112]. Metallic nanotubes allow nearly ballistic conduction along their axis, meaning electrons can travel with minimal scattering. In general, both SWCNTs and MWCNTs exhibit conductivities comparable to or exceeding copper, particularly when properly aligned. This phenomenon arises from the continuity of the π-conjugated network along the nanotube. Certain structural configurations (such as zigzag or chiral arrangements) can induce semiconducting behavior, further broadening their applications in nanoelectronics [112].

Carbon nanofibers (CNFs), on the other hand, constitute a distinct class of nanomaterials, formed by solid filaments with diameters ranging from the submicrometer to micrometer scale [114]. Their internal structure consists of turbostratically arranged graphite layers [116], differentiating them from the more ordered symmetry of CNTs. CNFs have a relatively high elastic modulus, ranging from 100 to 300 GPa [117], and a tensile strength of a few GPa [117], placing them below CNTs in mechanical performance. Nevertheless, they exhibit good electrical conductivity and are widely used as reinforcement materials in composites and as electrodes in electrochemical devices, where their cost-to-performance ratio is especially attractive.

### 4.5. Hybrid and Composite Materials

Hybrid or composite materials incorporate biomass-derived carbon structures with other components (metals, metal oxides, or polymers) to enhance their properties in environmental applications. In general, plant-based carbons provide high surface area and porosity, and when combined with metals or metal oxides, they act as excellent dispersing supports for active phases. This results in materials with functional synergies: for example, metal nanoparticles on carbon facilitate enhanced catalytic or adsorption reactions, while polymeric matrices combined with carbon yield more stable or responsive sorbents. The main types studied in the recent literature, with a focus on environmental applications and key findings, are described below.

#### 4.5.1. Carbon–Metal Composites

In these materials, metals (e.g., Fe, Co, Pd, Ni, Cu) are deposited or embedded in the biomass-derived carbon matrix, typically via chemical impregnation or hydrothermal treatments. These metals provide active catalytic sites for contaminant degradation or ion adsorption, while the carbon serves as a high-surface-area support. For instance, Masud et al. [118] demonstrated that iron-integrated biochar achieved highly efficient removal of organic contaminants via advanced oxidation processes: the presence of Fe generates reactive species (•OH, SO_4_•^−^, etc.) facilitating degradation, with performance significantly superior to unmodified biochar. Similarly, hydrogen storage performance improves when carbons are loaded with noble metals: for example, wood-derived carbons modified with Pd nanoparticles quadrupled H_2_ adsorption capacity (reducing the activation barrier) compared to the original material [119]. These results illustrate the metal–carbon synergy. Other metals such as Ni, Co, or Cu on plant-based carbons have been used for catalytic reactions (e.g., ammonia synthesis or contaminant hydrogenation) or to adsorb toxic metal ions [120,121,122]. In all cases, carbon–metal composites offer higher surface area and thermal stability than pure metals, enhancing efficiency in environmental remediation.

#### 4.5.2. Carbon–Metal Oxide Composites

In these hybrids, biomass-derived carbon is combined with metal oxide nanoparticles (e.g., ZnO, TiO_2_, Fe_3_O_4_, MnO_2_, MgO). The idea is to leverage the photocatalytic properties of the oxides alongside the high porosity of carbon. Recent studies confirm synergistic effects: incorporating ZnO, TiO_2_, or Fe_3_O_4_ into biochars enhances the photocatalytic and electrochemical activity of the pure oxide [123]. For example, Diacon et al. [124] synthesized nanocomposites from cherry pit-derived carbon with ZnO and Li_2_O, obtaining materials with improved absorption bands and effective degradation of organic dyes under UV. In adsorption applications, Ghandali et al. [125] reported that biochar impregnated with ZnO and MnO_2_ nanoparticles was highly effective for immobilizing heavy metals in contaminated soils, ZnO provides affinity for Pb^2+^ and Cd^2+^, while MnO_2_ offers –OH groups that complex these ions. Together, the carbon–oxide system enhances ion-exchange capacity and retains metals more efficiently than biochar or oxides alone. Biomass-derived carbon–oxide composites exhibit significantly higher degradation rates of organic contaminants (e.g., dyes, phenols) than bulk oxides [123]. Beyond photocatalysis, they are employed for peroxide activation and metal adsorption in wastewater, benefiting from enhanced porosity and new surface groups introduced by the oxide.

#### 4.5.3. Polymer–Carbon Composites

These materials combine biopolymers or synthetic polymers with biomass-derived carbons. For instance, polyaniline (PANI), polypyrrole (PPy), or other conductive polymer matrices integrated with activated carbon can create highly sensitive sensors or sorbents [126]. Luo et al. [127] incorporated activated carbon from coconut shells into a PANI film for H_2_S detection: the carbon increased active surface area and facilitated doping processes, resulting in a stronger and more stable sensor response. Another example is biocomposite materials based on natural polymers (alginate, cellulose, chitin) with biochar particles, used as adsorbents for metals and dyes. Overall, incorporating biomass-derived carbon into a polymer matrix provides additional adsorption sites and structural rigidity, improving the capture of environmental contaminants. Therefore, these polymer–carbon composites leverage the sustainability of plant-based carbon and the specific properties of the polymer (e.g., conductivity or biodegradability), resulting in high-performance, recyclable adsorbents.

## 5. Green Synthesis Strategies for Carbon Nanomaterials

### 5.1. Conventional Thermal Methods

#### 5.1.1. Pyrolysis

Pyrolysis is traditionally defined as the thermal decomposition of organic compounds under complete or partial absence of oxygen, where covalent bond cleavage is driven primarily by heat [128]. Owing to its versatility, pyrolysis has been widely adopted for the synthesis of carbon nanomaterials, as it enables the conversion of solid, liquid, and gaseous organic precursors into carbonaceous materials with tunable microstructures. In this context, systematic optimization of process parameters (such as precursor composition, decomposition pathways, temperature profile, and applied pressure) has been shown to govern the formation of one-dimensional and two-dimensional nanostructures, highlighting the strong dependence of material architecture on synthesis conditions [128].

Pyrolysis can be classified according to the physical state of the precursor, among which solids such as biomass, coke, coal, and municipal solid waste stand out, as well as liquids such as hydrocarbons or oils [128,129].

**Figure 4:**
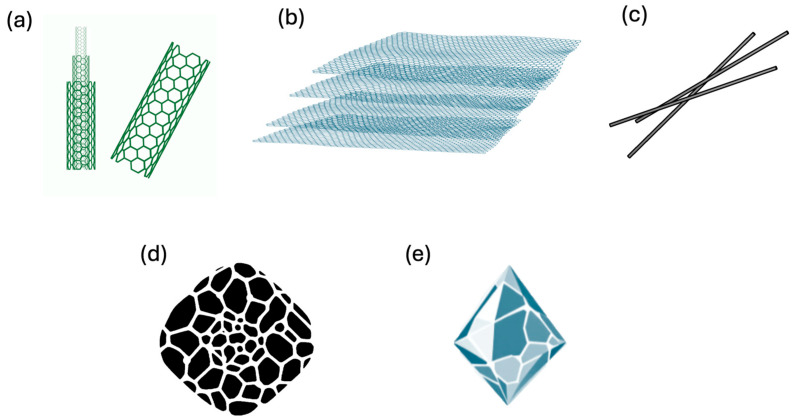
Main carbon nanomaterials produced by pyrolysis. (**a**) CNTs, (**b**) graphite, (**c**) carbon fibers, (**d**) glass-like carbon, and (**e**) diamond-like carbon (DLC).

According to Devi et al. [128] the main technologically significant nanomaterials produced through pyrolysis (Figure 4) include CNTs, carbon fibers (CFs), diamond-like carbon (DLC) films, glass-like carbon (GC), and graphite.

The biomass employed in the synthesis of porous carbon nanomaterials can originate from a wide variety of carbon-rich sources, including agricultural residues, shells, leaves, plant matter, and microorganisms, enabling the production of materials with diverse compositions and functionalities. In many cases, the intrinsic presence of heteroatoms within the biomass facilitates in situ doping during synthesis; for instance, nitrogen-rich feedstocks such as shrimp shells can yield nitrogen-doped carbon materials without the need for external dopant precursors [130]. Beyond precursor selection, the physicochemical properties of the resulting carbon nanomaterials can be systematically tuned by controlling key process parameters, including reaction time, temperature, and the use of activating or modifying additives during conversion [131].

#### 5.1.2. Carbonization

**Figure 5:**
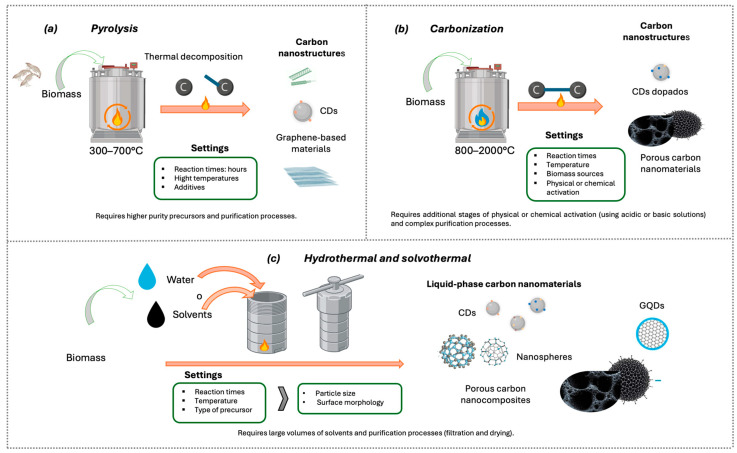
Schematic illustration of conventional thermal methods. (**a**) Pyrolysis process, (**b**) Carbonization process, and (**c**) Hydrothermal/solvothermal process.

Carbonization is a thermochemical process that, in contrast to pyrolysis (Figure 5), involves the formation and reorganization of C–C bonds and typically occurs at temperatures ranging from 800 to 2000 °C [128]. In biomass-derived routes, carbonization is commonly employed to decompose the organic constituents of the precursor and promote the development of carbon nanostructures [127,128,129,130,131,132]. This step is frequently coupled with subsequent physical or chemical activation treatments, using acidic or basic agents, to enhance porosity and tailor the surface chemistry of the resulting materials, thereby improving their functional performance [127,129,130,133].

The performance of advanced carbon materials derived from biomass is strongly dependent on the structural characteristics of the precursor [134]. Biomass with an inherently loose and porous architecture tends to yield carbon materials with enhanced electrochemical performance after carbonization and activation, as such structures facilitate pore development and ion transport [134].

### 5.2. Hydrothermal and Solvothermal Methods

The hydrothermal and solvothermal methods are widely used techniques for producing carbon nanocomposites in the liquid phase. These synthesis strategies are carried out in sealed reactors (Figure 5c), where carbon sources are subjected to heating in water or organic solvents to obtain carbon-based nanocomposites. When water is used as the reaction medium, the process is referred to as the hydrothermal method, while the use of organic solvents is known as the solvothermal method [135].

One of their main advantages is the ability to control properties such as particle size, surface morphology, and optical response by adjusting synthesis parameters including temperature, reaction time, and the type of carbon precursor employed [135]. The use of biomass as a carbon source has proven to be a sustainable and cost-effective approach, and several studies have achieved successful results in the synthesis of carbon dots (CDs) and other nanocomposites using these methodologies.

These techniques have demonstrated high efficiency in producing carbon dots, spheres, and porous nanomaterials [136,137,138].

In hydro/solvothermal synthesis, the comparatively low specific surface area is an inherent consequence of operating at moderate temperatures in the liquid phase, which limits extensive pore development. However, this apparent drawback is partially offset by the high density of oxygen- and nitrogen-containing surface groups typically introduced under these conditions. Such functionalities enhance affinity toward metal species and electrolytes, enable a rich surface chemistry (facilitating catalyst anchoring, the generation of active sites, and photoluminescent behavior), and favor applications in which surface functionalization plays a more decisive role than high surface area itself.

### 5.3. Assisted and Advanced Techniques

#### 5.3.1. Microwave-Assisted Synthesis

**Figure 6:**
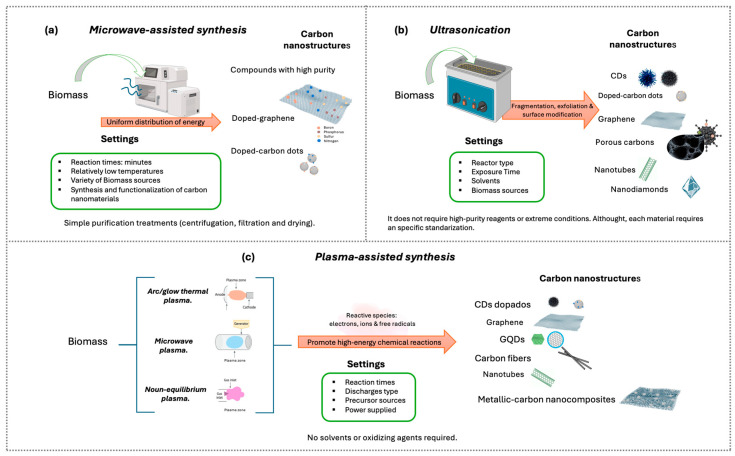
Schematic illustration of assisted and advanced techniques. (**a**) Microwave-assisted synthesis, (**b**) Ultrasonication, and (**c**) Plasma-assisted synthesis.

Microwave-assisted synthesis (Figure 6a) has emerged as an efficient strategy for producing carbon nanomaterials. Unlike the conventional methods described previously, microwave irradiation enables rapid and uniform heating of the materials, thereby accelerating chemical reactions [139,140]. This process may require only minutes, whereas other methods typically require several hours. The more homogeneous energy distribution promotes uniform particle sizes [135,140].

The relatively low operating temperatures favor the production of nanocomposites with high purity, large surface area, and remarkable adsorption capacity and facilitate the incorporation of functional reagents for heteroatom doping (e.g., boron, nitrogen, phosphorus, sulfur), as well as the processing of a wider variety of precursors, including biomass [135].

Carbon nanocomposites synthesized and/or functionalized using this technique have shown promising applications in the treatment of industrial and municipal wastewater [135,139,140]. Several studies have confirmed the usefulness of microwave-assisted synthesis for producing carbon nanomaterials from biomass. For example, in 2017, Regina et al. used Calamansi juice as a carbon source in combination with EDA to prepare carbon dots (CDs) [135].

**Figure 7:**
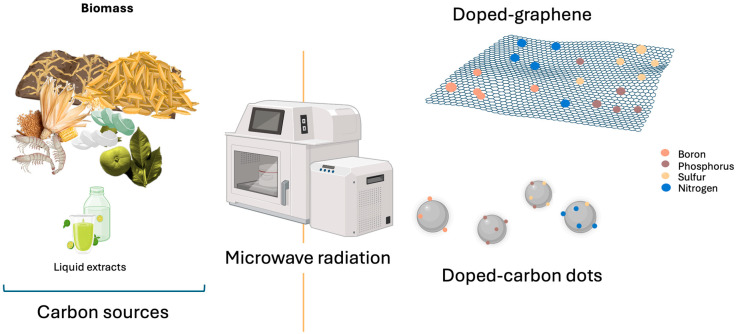
Heteroatom-doped nanomaterials synthesized by microwave-assisted methods.

Compared to other conventional methods, microwave-assisted synthesis is a non-invasive, simple, and rapid technique. The straightforward post-processing (centrifugation, filtration, and drying) makes it suitable for large-scale production [135]. This synthesis method has also enabled flexible control over the degree of reduction and exfoliation of graphene-based nanocomposites [141], as well as effective heteroatom doping (Figure 7).

Despite these advantages, microwave-assisted synthesis presents important limitations in terms of scalability and process reproducibility. The apparent uniformity of microwave heating is highly dependent on reactor design, precursor dielectric properties, and batch size and may deteriorate at larger scales, leading to thermal gradients, localized overheating, and batch-to-batch variability in material properties. Moreover, accurate temperature control under microwave irradiation remains challenging, which complicates process scale-up and limits the direct transfer of laboratory protocols to industrial production. As a result, while microwave-assisted synthesis is well suited for rapid and small-scale material fabrication, overcoming these constraints is essential for its reliable large-scale implementation.

#### 5.3.2. Ultrasonication

Ultrasonication (Figure 6b), also known as sonication, is a green technique that employs ultrasonic waves to induce acoustic cavitation in liquid media [140,142]. During this process, microbubbles are generated, which, upon violent collapse, produce extreme localized temperature and pressure conditions, accompanied by microjets and high-intensity shear forces. These conditions promote fragmentation, exfoliation, and surface modification of materials, facilitating the conversion of renewable precursors into carbon nanomaterials such as carbon dots (CDs), graphene, nanotubes, and nanodiamonds [142,143,144,145,146].

Ultrasonic treatment generates controlled surface damage (cracks, fractures, and cavities) in the precursors, increasing the available surface area for chemical or biocatalytic reactions [147]. Additionally, this mechanical action promotes functionalization and heteroatom doping (N, S, P), which modifies the graphite structure and the electronic properties of carbon nanomaterials [135,144,147]. For instance, Zhao et al. [143] reported the production of biomass-derived CNDs with good photostability and optical properties, without the need for high-purity chemical reagents or extreme conditions, demonstrating their viability as bacterial imaging agents.

The versatility of this technique is evident in the synthesis of functional nanomaterials such as fluorescent CDs, porous carbons, and hybrid materials. CDs with high quantum efficiency have been obtained from natural residues such as crab shells, *Polyalthia longifolia* leaves, cigarette ash, and dextrose, with applications in photocatalysis and environmental remediation [142,148]. Likewise, porous carbons derived from garlic peels [144] and binary nickel-cobalt oxides on activated biocarbon [120] have been reported, with applications as heavy metal adsorbents (Zn(II), Cd(II)) and in energy storage devices [120,148].

Several studies have shown that ultrasound promotes homogeneous dispersion of metal nanoparticles and preserves the porous structure of carbon supports. For example, Gado et al. [120] used an ultrasound-assisted synthesis method to uniformly disperse NiO and CoO nanoparticles in an activated biocarbon matrix, obtaining scalable and efficient materials for water remediation. In these cases, ultrasonic cavitation improves liquid penetration into the carbon pores, removes impurities, and facilitates dopant incorporation.

The effect of ultrasonic treatment strongly depends on the type of reactor, power, exposure time, and the nature of the solvents and substrates used [146]. Although this technique reduces reaction times and promotes the formation of fine particles, each material requires specific optimization. To date, most studies have been carried out at a small scale [146].

#### 5.3.3. Plasma-Assisted Synthesis

Plasma-assisted synthesis relies on the generation of a partially ionized medium composed of reactive species such as electrons, ions, and free radicals, which promote high-energy chemical reactions without the need for solvents or oxidizing agents [135]. Its versatility allows tuning the system’s energy and directing the synthesis toward different morphologies, such as quantum dots, graphene, nanotubes, and metal–carbon nanocomposites [135,149,150] (Figure 6c).

Depending on the type of discharge used to generate the plasma, cold atmospheric plasma, thermal arc or discharge plasma, and microwave plasma can be distinguished, each with particular characteristics of temperature, energy density, and types of reactive species [135].

The plasma environment enables surface functionalization and heteroatom doping (N, S, B), modulating the electronic and catalytic properties of the synthesized materials [149,150,151,152]. For example, Ma et al. reported N-doped CDs using microplasma [135]. Additionally, metal–carbon nanocomposites have been effectively synthesized, such as N-doped Pd/AC (activated carbon) [150] or PtFe@NCNT-P with N-doped carbon layers [151], as well as graphene quantum dots (GQDs) with fine particle size control and Ag–C nanocomposites with high crystallinity and nanoparticle size control [135,149].

The use of biomass as a carbon source in these systems represents a sustainable alternative to fossil precursors. Qasim et al. reported the production of carbon nanotubes and fibers from wood sawdust via plasma-assisted pyrolysis [153], while Nandihalli employed organic waste to synthesize multilayer graphene and CNTs in very short reaction times [153]. In both cases, plasma simultaneously promoted carbonization, doping, and functionalization, producing materials with high purity and enhanced properties.

Plasma is considered one of the most promising strategies in the green synthesis of carbon nanocomposites, although challenges remain related to parameter standardization, uniformity of doping, and process scalability.

#### 5.3.4. Hybrid/Combined Methods

**Figure 8:**
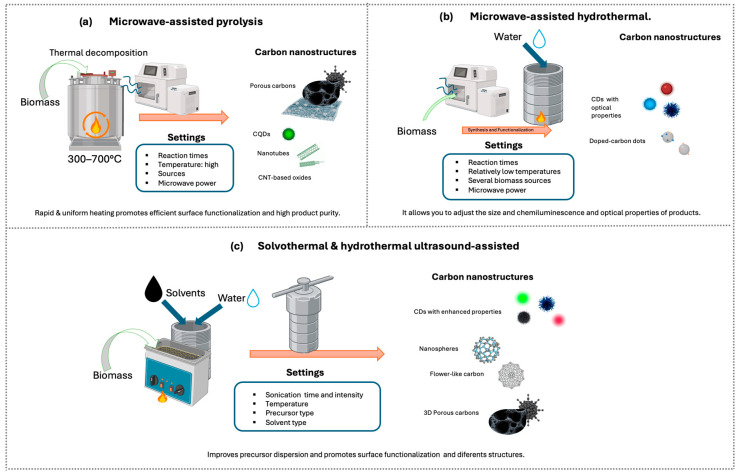
Schematic illustration of hybrid methods and main CNMs. (**a**) Microwave-assisted pyrolysis, (**b**) Microwave-assisted hydrothermal, (**c**) Solvothermal and Hydrothermal ultrasound-assisted.

Hybrid methods combine different synthesis techniques to leverage the advantages of each and obtain nanomaterials with improved properties in shorter times and with greater control over morphology, size, and functionalization. Among the most studied combinations are microwave- and ultrasound-assisted techniques, in conjunction with pyrolysis, hydrothermal, or other synthetic routes [146,148] (Figure 8). The use of hybrid methods in the synthesis of carbon nanomaterials from biomass has shown particular efficacy due to the ability to activate the carbon source and/or promote functionalization.

The combination of ultrasonication with other synthetic routes, such as pyrolysis, has shown significant results, enhancing both the functionalization and yield of carbon nanocomposites [146,148]. For example, ref [146] discusses the preparation of three-dimensional hierarchical porous carbons from garlic peels via ultrasound-assisted impregnation, achieving homogeneous bimetallic activation and an interconnected pore network. Furthermore, ultrasound-assisted hydrothermal synthesis improves precursor dispersion and nucleation, producing CDs with enhanced optical properties, such as those reported by Usman and Cheng, with quantum yield (QY) up to 40.5%, high optical stability, and photodegradation resistance, or highly stable and photodegradation-resistant CDs produced from whole wheat bread and natural additives [120,142,154].

Similarly, microwaves are also used in combination with other methods. For instance, Ji et al. employed a combined microwave and hydrothermal treatment to prepare near-infrared (NIR) CDs from *o*-Phenylenediamine (*o*-PDA) as a precursor. This combination accelerated the synthesis, improved yield, and promoted surface functionalization of the CDs [135]. Park et al. obtained 2–4 nm CDs with deep ultraviolet or blue emission and QY up to 11% from glucose in just a few minutes. Similar treatments with glycerol, sucrose, or polyethylene glycol (PEG) have enabled tuning the particle size and chemiluminescence of the products [154]. Microwave-assisted pyrolysis of carbohydrates and other small molecules in aqueous media has also been shown to produce soluble CDs with high yields [154].

### 5.4. Comparison of Methods

Among the most employed approaches in the green synthesis of carbon nanomaterials from biomass are pyrolysis, hydrothermal carbonization (HTC), microwave-assisted synthesis, and ultrasonic irradiation. These methods offer environmental and operational advantages compared to other chemical processes [137,140,155].

Pyrolysis has been widely used for the synthesis of carbon quantum dots (CDs) and graphene-based materials [128,148]. This method produces high-purity carbon with good crystallinity and porosity; however, it requires high temperatures (500–800 °C) and strict process control, implying high energy consumption and the need for dry biomass. In contrast, hydrothermal carbonization (HTC) is distinguished by its ability to directly convert wet biomass into carbonaceous materials, using water as the reactive medium under moderate temperature and pressure conditions, which significantly reduces energy requirements [4,140].

Both hydrothermal and solvothermal synthesis allow fine control over the size and morphology of nanomaterials by adjusting parameters such as temperature, time, and precursor concentration. In hydrothermal synthesis, the aqueous medium facilitates the formation of uniform carbon structures, whereas in solvothermal approaches, organic solvents such as ethanol, acetone, or dimethylformamide modulate the polarity and degree of carbon reduction, promoting surface functionalization [137,155].

Microwave- and ultrasound-assisted techniques, on the other hand, represent highly efficient emerging alternatives. The rapid and uniform heating produced by microwave irradiation reduces reaction times to a few minutes and promotes homogeneous conversion of biomass into carbon nanomaterials. This method has proven effective for transforming biomass such as rice husks, sago residues, shrimp shells, or vegetable oils into heteroatom-doped CDs and CNTs with high purity and low cost [140,145]. Additionally, ultrasonication has been shown to facilitate carbon functionalization, outperforming solvothermal and microwave-based routes significantly [146].

Biomass-based processes also offer remarkable versatility in the carbon sources used, ranging from plant waste (leaves, fruits, lignin, cellulose, used paper) to animal residues (keratin, fish scales, crustacean shells). These raw materials contain heteroatoms such as N, S, and O, which facilitate the formation of doped nanomaterials without the need for external chemical agents, enhancing the photoluminescence and biocompatibility of the final products [140].

Comparatively, HTC and microwave-assisted methods are the most consistent with the principles of green chemistry, allowing the use of wet biomass, short reaction times, and lower energy consumption. In contrast, pyrolysis, although effective for producing graphitic carbon, requires more energy and exhibits lower efficiency. Ultrasonic irradiation and plasma treatments emerge as promising routes for obtaining functional nanocomposites under environmentally friendly conditions, pointing toward the development of cleaner, scalable, and economically viable technologies.

These techniques not only diversify the synthetic routes toward carbon nanomaterials but also consolidate biomass as a strategic precursor for the sustainable production of advanced materials with diverse applications.

Table 2 summarizes the main characteristics of methods for synthesizing carbon nanomaterials from biomass, highlighting reaction times, the nature of post-treatments and the potential for scaling associated with each technique.

### 5.5. Interplay Between Synthesis Parameters, Activation Mechanisms, and Material Properties

The physicochemical properties of biomass-derived carbon nanomaterials are strongly governed by the interplay between synthesis parameters, including temperature, activation strategy, and precursor composition [100]. Among these factors, the activation mechanism plays a decisive role in defining pore architecture, surface chemistry, and the degree of structural ordering, which in turn determine material performance in specific applications.

Chemical activation, particularly using alkali agents such as KOH, typically promotes extensive microporosity and high specific surface areas through redox reactions and carbon etching processes occurring at elevated temperatures. This approach often yields highly developed pore networks with narrow pore size distributions, which are advantageous for applications requiring high adsorption capacity, such as gas capture or the removal of small organic pollutants [161]. However, the aggressive nature of chemical activation may also lead to partial collapse of the carbon framework, reduced structural integrity, and increased environmental burdens associated with chemical consumption and post-treatment washing steps.

In contrast, physical activation using oxidizing gases such as CO_2_ or steam proceeds through more gradual gasification reactions, generally producing broader pore size distributions with a higher contribution of mesoporosity [162]. Although this method typically results in lower surface areas compared to chemical activation, the improved pore accessibility and reduced diffusion limitations are particularly beneficial for the adsorption of larger molecules and for applications involving rapid mass transfer, such as wastewater treatment and catalytic processes. Moreover, physical activation avoids the use of corrosive chemicals, offering advantages in terms of process simplicity and environmental compatibility, albeit at the expense of higher energy demand due to longer activation times and higher operating temperatures. A comparative overview of the main synthesis and activation routes, highlighting their typical temperature ranges, dominant pore structures, surface functionalities, and key advantages and limitations, is provided in Table 3.

Beyond the activation strategy, different parameters (such as pyrolysis temperature or biomass composition) further modulate surface functionalization and the degree of graphitic ordering. Lower temperatures preserve oxygen-containing functional groups that enhance polarity and surface reactivity, while higher temperatures favor aromatic condensation and partial graphitization, improving electrical conductivity and hydrophobic interactions [163]. Consequently, no single synthesis route can be considered universally optimal; instead, the selection of synthesis and activation parameters must be tailored to balance porosity, surface chemistry, and structural order according to the targeted application.

## 6. Structure–Property–Performance Relationships in Environmental Applications

### 6.1. Role of Surface Chemistry and Functional Groups

The types and densities of surface functional groups on biomass carbon critically govern adsorption. Acidic oxygenated groups (–COOH, –OH, phenols) and heteroatoms (e.g., N, S) serve as active binding sites. For heavy metals, chemisorption via complexation and ion exchange dominates; carboxylates and phenolics coordinate metal cations and even reduce Cr(VI) to Cr(III), acting as hard Lewis bases, whereas aromatic carbonyl or π-sites enable soft-binding (e.g., cation–π) [164,165]. Nitrogen dopants (pyridinic, pyrrolic, amine) on biochar markedly enhance metal uptake by providing electron lone pairs for chelation [165].

In contrast, organic molecules interact primarily through π–π and hydrogen-bonding interactions: π-conjugated graphitic regions of the carbon enable π–π stacking with aromatic rings (dyes, antibiotics, phenols), while polar groups on both adsorbent and adsorbate form H-bonds or electrostatic links. For example, cationic dyes (e.g., methylene blue) are adsorbed strongly by negatively charged surface sites and π–π forces, whereas anionic dyes (e.g., methyl orange) depend more on pore-filling and H-bonding [166]. Similarly, polar pharmaceuticals like tetracycline exhibit mixed-mode uptake (van der Waals, H-bonding, π–π and ionic interactions) [167]. In sum, surface chemistry dictates whether adsorption is dominated by ionic complexation or by hydrophobic/π interactions, and tuning of –COOH/–OH vs. aromatic content can “switch” the affinity toward metals versus organic contaminants [164,168].

### 6.2. Influence of Porosity and Surface Area

Porous texture largely controls capacity and kinetics. High BET surface area correlates with higher overall uptake because more sites are available [169]. Micropores (<2 nm) provide the greatest surface area per mass and thus contribute most to adsorption capacity for small molecules and ions; in fact, the volume of micropores often tracks pollutant uptake [169]. Mesopores (2–50 nm) and macropores primarily serve as diffusion highways that allow large molecules (e.g., bulky dyes, antibiotics) to reach interior sites. For instance, KOH-activated carbons from biomass can achieve >1000 m^2^/g with hierarchical micro/mesoporosity, combining high capacity (via abundant micropores) with faster kinetics (via mesopores). This is evident in dye adsorption: mesopore-rich carbons facilitate pore-filling of large dye molecules, whereas micropore-rich carbons maximize adsorption of smaller cationic dyes [166]. In practice, the activation method tunes the pore architecture (e.g., steam/CO_2_ activation yields primarily micropores, whereas alkali treatment yields more mesopores). Thus, performance is governed by matching pore size to pollutant size: microporous biochars excel at capturing small ions and nonpolar organics, while hierarchical carbons ensure fast uptake of larger organics and dyes.

### 6.3. Graphitic Domains and Defect Density

The degree of graphitization (sp^2^ aromatic content) and defectiveness profoundly affect adsorption mechanisms. Highly graphitic carbons (formed at high pyrolysis temperature) have extended π-conjugation and low polar functionality. Such materials strongly promote π–π donor–acceptor interactions and hydrophobic adsorption of aromatic pollutants [168]. For example, high-temperature biochar with condensed aromatic surfaces exhibits stronger π–π stacking with nonpolar aromatics than low-temperature chars [168]. However, increased order generally comes at the expense of surface heteroatoms: graphitic regions lack the –OH/–COOH binding sites needed for metal complexation, so extremely ordered carbons may perform worse for metal uptake. By contrast, defect sites (edges, vacancies, heteroatom dopants) introduce localized polarity and chemisorptive centers. Nitrogen or oxygen at defects acts as Lewis bases or coordinators, so a defect-rich carbon with pyridinic/pyrrolic N and residual –OH groups binds metals strongly [165]. In practice, one aims for an optimal balance: graphitic domains maximize π-interactions for aromatic organics, while defects and heteroatoms supply electron donors for ionic binding. As such, performance is *governed* by the interplay of conjugated sp^2^ planes and the density of chemically active defect sites [164,165].

### 6.4. Implications for Different Classes of Pollutants

These structure–property principles manifest uniquely for each pollutant class. Heavy metals: Removal is dominated by surface complexation and ion exchange with acidic or basic surface groups. Biomass carbons rich in –COOH/–OH (often from moderate pyrolysis/oxidation) and N-sites (via doping or proteinaceous biomass) show the highest uptake [164,165]. Dyes: Cationic dyes are captured by electrostatic attraction to negatively charged (deprotonated) groups and by π–π stacking, whereas anionic dyes rely on hydrogen bonding, π interactions, and pore entrapment [166]. Thus, carbons with abundant surface charges and aromatic regions (e.g., acidic activated biochar) are most effective. Antibiotics (e.g., tetracyclines, fluoroquinolones): These polar-aromatic molecules engage multiple interactions (π–π, H-bond, ionic), so high-surface-area carbons with tailored functionality (e.g., N-doped, tunable pH sites) perform best [167]. Aromatic organics (phenols, endocrine disruptors, PAHs): Uptake is driven by π–π donor–acceptor and hydrophobic partitioning. Thus, highly aromatic, hydrophobic carbons (from high-temperature pyrolysis) with extensive microporosity excel [168]. In all cases, hierarchical porosity improves kinetics for bulky molecules and enables facile transport into active sites.

Table 4 summarizes these relationships. (Notably, similar trends apply to gaseous pollutants: e.g., CO_2_ adsorption is enhanced by basic sites and microporosity, reflecting the same structure–performance drivers.). This overview highlights how specific material properties, derived from tailored synthesis and activation strategies, control the primary removal mechanisms for metals and organic pollutants.

## 7. Environmental Applications

### 7.1. Adsorption of Heavy Metals and Organic Pollutants

Adsorption is one of the most widely used strategies for removing contaminants (heavy metals and organic compounds) using bio-derived nanomaterials. Activated carbon adsorbents derived from biomass offer high surface area, hierarchical porosity, and high adsorption capacity [170]. For example, it has been observed that nanotubes and activated carbon obtained from agricultural waste, after activation or oxygen-group doping treatments, can capture metals such as Pb^2+^, Cd^2+^, or Cu^2+^, among others, through chemical and physical interactions [171]. The adsorption mechanisms include physical adsorption (Van der Waals forces), electrostatic interactions (positively charged ions attracted to negatively charged surface sites), surface complexation with functional groups (carboxyls, phenols), and ion-exchange processes [171].

Key parameters defining performance include pH, adsorbent dosage, and contact time. In practice, maximum adsorption capacities (q_max) can be very high: q_max values of tens to hundreds of mg of heavy metal per gram of adsorbent have been reported. For example, Okon-Akan and collaborators modified a biochar that initially removed 92% of Cd and 80% of Pb, equivalent to ≈15 and 34 mg/g, respectively; after regeneration, it reached q_max values of ~92 mg/g (Cd) and ~272 mg/g (Pb) [172]. Similarly, the adsorption of organic contaminants (such as dyes, pharmaceuticals, or pesticides) by biomass-derived carbon is often highly efficient. It has been reported that modified biomass adsorbents are preferred due to their large surface area, which enhances the capture of organic molecules compared to other materials. For instance, activated adsorbents from plant waste can remove synthetic dyes with near 100% efficiency under optimal conditions [159]. Heavy metal removal mainly occurs through electrostatic interaction, complexation, ion exchange, reduction, and hydrogen bonding. For organic contaminants, predominant mechanisms include pore filling, π–π interactions, hydrophobic effects, and electrostatic attraction.

### 7.2. Photocatalysis and Advanced Oxidation Processes

Biomass-derived carbon nanomaterials also enhance photocatalytic and advanced oxidation processes for contaminant degradation. A notable case involves carbon quantum dots (CDs) obtained from agricultural residues (e.g., fruit peels or bagasse), which absorb visible or UV light and facilitate photochemical reactions. Chávez-García et al. synthesized CDs from grape pulp and watermelon rind that, under sunlight, effectively degraded the methylene blue dye in a short period [173]. These CDs exhibited optical properties (upconversion/downconversion fluorescence) and diverse surface groups, enabling them to act as low-cost, sustainable catalysts for removing industrial dyes [173].

Additionally, biomass-derived carbon can serve as a support or co-catalyst for conventional semiconductors. Biochar and nanocarbons can coat or be combined with TiO_2_, ZnO, and other photocatalysts. This coupling offers several advantages: biochar can reduce the bandgap of TiO_2_ (extending its absorption to broader wavelengths), and its high conductivity facilitates the transfer of photogenerated electrons, thus reducing electron–hole recombination. Specifically, supporting TiO_2_ on biochar decreases the bandgap and expands the UV–Vis absorption range [174]; additionally, the delocalized π electrons in biochar promote charge separation. Oxygen-containing functional groups and persistent free radicals on the biochar surface can also activate oxidants (e.g., H_2_O_2_) to generate •OH radicals capable of degrading contaminants [174]. As a result, hybrid biochar/semiconductor systems often achieve higher degradation efficiencies than pure photocatalysts. For example, coffee-derived biochar/TiO_2_ composites have demonstrated enhanced phenol removal due to phenolic functionalities that facilitate electron transfer [174]. Overall, the use of biochars and carbon dots (CDs) in photocatalytic processes and heterogeneous Fenton systems significantly improves the degradation of recalcitrant organic compounds, making these processes more sustainable.

### 7.3. Electrochemical Applications in Water Treatment

Biomass-derived nanocarbons are also employed in electrochemical technologies for water purification. In capacitive deionization (CDI), porous electrodes store salt ions when a voltage is applied. Recent studies highlight that carbonaceous materials derived from biomass are promising candidates for CDI due to their abundance, low cost, and tunable structures [175]. Jin-Jing Jiang et al. report that the desalination performance of CDI depends critically on the electrode material; biomass-derived carbons offer high surface area and conductivity, which are ideal for adsorbing large quantities of ions [175]. Although commercial activated carbon is efficient, its production may have environmental impacts, which has motivated the investigation of sustainable alternatives such as biochars, nanotubes, or graphene obtained from agricultural residues (e.g., coconut shell, sugarcane bagasse). These porous structures typically exhibit high surface capacitance, though their charge storage capacity may be limited, encouraging modifications (e.g., doping or forming composites with metals) to enhance ionic performance.

Another relevant electrochemical route is electrocatalytic contaminant degradation. For example, biomass-derived carbon materials can be used in electro-Fenton processes: the electrode catalyzes the reduction of O_2_ to H_2_O_2_ (via a two-electron pathway), which, together with Fe^2+^ in solution, generates hydroxyl radicals to oxidize contaminants. Wen et al. prepared a carbon material from pyrolyzed Spirulina at 500 °C (HBC-500), and after surface modification, it electrochemically produced 238.4 mg/L of H_2_O_2_ in 90 min at 50 mA/cm^2^. Under optimal conditions, this bio-carbon achieved >95% degradation of model organic pollutants within just 30 min (pH 5). Furthermore, after multiple cycles, it maintained >91% activity, demonstrating good stability [176]. These results indicate that biomass-derived carbons (e.g., algae-based or modified wood carbons) can serve as economical and efficient electrocatalysts to generate reactive oxidizing species in situ and degrade toxic substances in wastewater.

Beyond electrocatalytic routes focused on contaminant degradation, the same structural and electronic features that enable efficient electro-Fenton processes in biomass-derived carbon materials (such as high electrical conductivity, tunable surface chemistry, and the presence of defect-rich or heteroatom-doped domains) suggest their potential applicability in other emerging electrochemical reactions. Recent fundamental studies on electrochemical CO_2_ conversion and C–N coupling reactions have demonstrated that carbon-based catalysts with tailored active sites can effectively mediate complex multi-electron transfer pathways, achieving high selectivity toward value-added products such as CO, formate, or urea [177,178]. While these investigations are not yet centered on biomass-derived carbons as mature electrocatalysts, they provide a mechanistic framework indicating that structurally engineered bio-based carbon nanomaterials could, in principle, participate in advanced electrocatalytic processes beyond water treatment, although further optimization and validation are required.

### 7.4. Sensing of Environmental Contaminants

Biomass-derived carbon nanomaterials are also used in electrochemical and optical sensors for environmental contaminant monitoring. In electrochemical sensors, carbons obtained from waste sources (e.g., wood, shells, cellulose) can serve as sensitive electrodes after chemical activation, doping, or combination with metal nanoparticles. Zhang et al. recently reviewed that these renewable and low-cost materials can be converted into high-performance electrodes by improving their porosity, conductivity, and wettability through treatments such as KOH activation, nitridation, or incorporation of metallic compounds [176]. As a result, these sensors can effectively detect biological molecules (e.g., glucose), heavy metal ions (Pb^2+^, Hg^2+^, Cd^2+^), and pesticide residues with high sensitivity and low detection limits [179].

Complementarily, optical sensors based on carbon quantum dots (CDs) enable rapid contaminant detection through fluorescence changes. CDs are chemically stable, highly luminescent, and water inert, making them versatile for on-site detection of various molecules. Studies have shown that CDs interact efficiently with pathogens, metal ions, and pesticides, producing fluorescence signals that shift in response to the presence of contaminants. For example, functionalized CD sensors have achieved ultrasensitive detection of Pb^2+^ and Hg^2+^ at picomolar levels [158]. Therefore, the combination of low toxicity, high solubility, and favorable photoelectric characteristics positions biomass-derived CDs as promising tools for both electrochemical and optical environmental monitoring.

### 7.5. Other Emerging Environmental Applications

In addition to the applications described above, new environmental uses of biomass-based nanomaterials are currently being explored. These include CO_2_ capture and conversion using activated biochar as sorbents, removal of volatile organic compounds through air filtration systems, and rehabilitation of degraded soils. For example, the production of biochar from agricultural residues has been proposed as a strategy for reducing greenhouse gas emissions and mitigating climate change [180]. Likewise, carbon nanoparticles and enriched biochar are being tested as additives in soil bioremediation systems (improving retention of toxic contaminants) and in microbial fuel cells for sustainable energy generation. Although these applications are promising, most are still in early development stages, and further studies are required to optimize both materials and processes.

## 8. Challenges and Future Perspectives

Biomass-derived carbon nanomaterials offer promising applications for environmental remediation (e.g., contaminant adsorption, photocatalysis, sensing) due to their renewable origin, low cost, and high surface activity. However, large-scale deployment presents environmental and safety challenges. For instance, although biochar provides high surface area and functional groups favorable for capturing toxic compounds, it can also act as a secondary source of pollution. When applied to soil or water systems, biochar may release heavy metals (Pb, Cr, Cd) and persistent organic pollutants (PAHs) originally present or formed during its production [181]. Additionally, persistent free radicals (EPFRs) have been identified on the biochar surface, capable of generating reactive oxygen species that can damage plant and animal cells [182]. These findings emphasize the need for comprehensive ecotoxicological evaluation before widespread implementation. Recent studies indicate that research on potential adverse environmental and health effects of biochar remains limited, leaving a critical knowledge gap [181].

### 8.1. Environmental and Toxicological Assessment

In the context of environmental applications, the environmental and toxicological assessment of biomass-derived carbon nanomaterials is essential. Although these nanomaterials have shown great potential for contaminant remediation, their compatibility with ecosystems must be verified through rigorous risk evaluation. This requires analyzing their physicochemical behavior in different media, as well as their environmental persistence and potential for bioaccumulation in living organisms.

Several recent studies indicate that many biomass-derived nanocarbons exhibit low intrinsic toxicity [183]. For example, carbon materials chemically obtained from carbon–organic aerosols have been evaluated in cell-based assays, showing high biocompatibility and no significant cytotoxicity [184]. These results suggest that, under controlled laboratory conditions, such nanomaterials do not cause evident cellular damage and could even be explored for biomedical applications (such as bioimaging) due to their low intrinsic toxicity [185].

Despite the favorable in vitro evidence, it is necessary to extend evaluation to realistic ecological scenarios. Toxicological tests using model organisms are key tools: for instance, assays with daphnia, fish, algae, or terrestrial invertebrates can reveal chronic or sublethal effects that may not appear in cell cultures. To date, some studies have reported that very high concentrations of certain nanocarbons may induce mild effects (e.g., on growth or reproduction rates) [186,187], although such levels are significantly higher than those expected under typical environmental application conditions.

Additionally, the final fate of nanocarbons after use must be considered. In bioremediation processes, these particles often act as adsorbents of heavy metals or other contaminants; therefore, it is crucial to ensure that they do not act as vectors that transfer such toxic substances into organisms within the food chain [156]. Environmental factors such as pH, sunlight exposure, or microbial activity can alter the chemical structure or aggregation state of nanocarbons [188], which in turn affects their degradation and environmental persistence. Thus, it is essential to investigate how these factors influence the stability and final disposition of the material after application.

Overall, the environmental and toxicological assessment of biomass-derived carbon nanomaterials is a necessary requirement for their safe implementation. Current evidence suggests that many of these nanocarbons exhibit low toxicity and good intrinsic biocompatibility, which aligns with their sustainable nature and eco-friendly applications. However, ensuring their complete safety requires systematic testing across multiple environmental matrices and representative organisms, following regulatory protocols and appropriate safety criteria to confirm the safety of these green technologies.

### 8.2. Research Directions and Opportunities

The optimization of sustainable processes for converting biomass into carbon nanomaterials represents a key step toward ecologically and economically viable alternatives. Methods such as pyrolysis, hydrothermal carbonization, and chemical activation have become promising routes for obtaining materials with high porosity, thermal stability, and abundant surface functionalization. However, standardizing these methods remains challenging, as factors such as biomass source, processing temperature, residence time, and activating agents can significantly influence the structural and chemical properties of the final product.

Moreover, the synthesis of advanced nanocomposites (such as biochar doped or functionalized with transition metals (Fe, Cu, Ni, Co) or semiconductors (TiO_2_, ZnO, g-C_3_N_4_)) opens new perspectives for designing multifunctional materials capable of acting in photocatalysis, water disinfection, and selective metal ion capture. These approaches combine the high surface area of carbon with the redox and photoactive properties of inorganic components, resulting in hybrid materials with strong potential for large-scale environmental applications.

In parallel, computational modeling and artificial intelligence (AI) have become fundamental tools to accelerate the development of biomass-derived carbon nanomaterials through rational design strategies. These methodologies enable the analysis of large volumes of experimental data and allow the prediction of structural and functional material properties prior to synthesis. Recent studies have demonstrated the effectiveness of machine learning (ML) for optimizing the production of biochar synthesized through pyrolysis and activation (porous carbon materials with wide technological applicability) [189]. ML models based on pyrolysis and activation conditions have accurately predicted porosity parameters such as specific surface area, total pore volume, micropore volume, mesopore volume, and average pore size. Experimental validation confirms the strong potential of machine learning for designing and optimizing high-porosity biochar, promoting more sustainable, reproducible, and efficient synthesis methodologies.

The “material mining” approach applied to biomass represents a decisive step toward data-driven innovation in materials science. By integrating physicochemical models, molecular simulations, and neural networks, it is possible to map the chemical diversity of biogenic sources and select those with the highest potential to generate high-performance carbon structures. This computational paradigm supports the creation of customizable materials with properties tailored to specific applications, such as photocatalysis, selective contaminant adsorption, or metal ion capture. Consequently, the convergence of green synthesis and artificial intelligence is redefining how nanomaterials are conceived, designed, and optimized, enabling scalable, sustainable, and predictive developments in biomass utilization.

### 8.3. Comparison with Fossil-Derived Carbon Materials and Life Cycle Considerations

A balanced assessment of biomass-derived carbon nanomaterials must explicitly recognize that the use of a renewable precursor does not automatically translate into a lower environmental footprint. Comparative life cycle assessment (LCA) studies indicate that biomass-derived activated carbons and biosorbents can exhibit comparable or, in some cases, lower impacts in categories such as global warming potential (GWP) and cumulative energy demand when compared to fossil-derived activated carbons; however, these outcomes are highly dependent on the activation route and process parameters employed. Meta-analyses of LCA studies report that biochar produced from agricultural residues may display lower GWP and energy demand than conventional activated carbons, even when transportation and production stages are considered, with results strongly influenced by the availability of renewable energy sources and the adoption of low-emission activation strategies [189]. Similarly, direct comparisons between biochar and biomass-derived activated carbons show that biochar production generally requires less energy and results in lower greenhouse gas emissions per kilogram of adsorbent, whereas the use of chemical activating agents such as H_3_PO_4_ or KOH can substantially increase environmental impact indicators [189]. Importantly, even under favorable LCA scenarios, biomass-derived carbons are not universally superior across all metrics: emissions associated with pyrolysis and activation steps may approach those of fossil-based carbons if energy inputs are not optimized and liquid or solid wastes are not properly managed. These observations highlight inherent trade-offs between sustainability and functional performance, underscoring the need for standardized and comparative LCA studies that integrate technical performance with environmental impacts across the entire life cycle and for careful consideration of energy sources, activation agents, and post-treatment strategies to genuinely optimize the sustainability of biomass-derived carbon materials.

## 9. Conclusions

Biomass-derived carbon nanomaterials constitute a promising platform for environmental applications, not only because of their renewable origin and low cost, but primarily due to the possibility of tailoring their physicochemical properties through controlled synthesis and activation parameters. This review highlights that the performance of these materials in contaminant removal (including heavy metals, pharmaceutical compounds, and dyes) is directly governed by surface chemistry, porosity, and the degree of structural order, rather than being incidental. In this context, understanding the relationships between synthesis parameters, resulting material properties, and removal mechanisms emerges as a central axis for the rational design of biomass-derived carbon materials.

Nevertheless, the environmental potential of biomass-derived carbons must be assessed critically. Although the valorization of biomass waste aligns with circular economy principles, certain synthesis and activation routes may involve high energy consumption or lead to the generation of undesirable byproducts, such as polycyclic aromatic hydrocarbons or residual metals, which pose environmental and health risks. In addition, the intrinsic heterogeneity of biomass precursors and process variability can limit reproducibility and, in some cases, performance relative to fossil-derived carbons, highlighting real trade-offs between sustainability and functional performance.

Future progress in this field will depend on integrated strategies combining rational material design, optimization of synthesis pathways, and systematic environmental assessment of the resulting nanomaterials. The development of safe-by-design materials, together with standardized methodologies and comprehensive life cycle assessment studies, will be essential to ensure that technological benefits translate into genuinely sustainable solutions. Ultimately, the conversion of biomass waste into functional carbon nanomaterials represents not only a technological opportunity but also a meaningful step toward environmentally responsible remediation strategies grounded in scientific rigor.

## Figures and Tables

**Table 1:** Recent progress and milestone achievements during 2012–2024 in the fabrication of different varieties of carbon-based nanostructures from biomass and their applications for new technologies.

S.N	Sources of Biomass	Conversion Method	Type of Nanostructure	Application and Year	Ref.
1	Bacterial cellulose	Pyrolysis/Carbonization	3D carbon nanofiber aerogels	Supercapacitors, batteries (2016)	[55]
2	Agricultural residues (rice straw)	Hydrothermal carbonization + CVD	Carbon nanotubes	Electrodes, catalysis (2017)	[56]
3	Natural alginate (algae)	Pyrolysis with Co/N doping	N-doped mesoporous carbon nanofibers	Li-ion batteries, ORR (2015)	[57]
4	Mushroom (enoki)	High-temperature pyrolysis	N-doped carbon	Oxygen reduction catalysis (2015)	[58]
5	Hemp stalk	Chemical activation + hydrothermal treatment	3D activated carbon + MnO_2_ nanowires	Supercapacitors (2017)	[59]
6	Cellulose/lignin	Direct carbonization	Carbon fibers and nanofibers	Purification, storage (2016)	[60]
7	Plant stems and leaves	Direct pyrolysis	Porous carbon	Supercapacitors (2016)	[61]
8	Various biomass	Pyrolysis, hydrothermal carbonization	Nanostructured porous carbons	Li–S batteries (2016)	[62]
9	Diverse biomass	Green synthesis, carbonization	Various carbon nanomaterials	Energy and environmental remediation (2016)	[63]
10	Various biomass	Pyrolysis, hydrothermal carbonization	Porous carbons	Batteries, supercapacitors (2016)	[64]
11	Biomolecules	Hydrothermal carbonization	Carbon nanospheres	Catalysis, encapsulation (2015)	[65]
12	Vegetable oils, plant residues	Pyrolysis, CVD	Carbon nanotubes	Electronics, sensors (2017)	[66]
13	Soy residues	Easy carbonization and activation	Nitrogen–oxygen co-doped porous carbon with honeycomb structure	Lithium–sulfur batteries (2016)	[67]
14	Rice husk	Direct synthesis	Silica-supported carbon quantum dots	Bioimaging, sensors (2017)	[68]
15	Various biomass	Pyrolysis, CVD, activation	Nanotubes, graphene, fibers	Agriculture, environment (2016)	[69]
16	Various biomass	Chemical activation	Nanoporous carbons	Batteries, supercapacitors (2012)	[70]
17	Corn cobs	NH_3_ activation	N-doped microporous monoliths	Selective CO_2_ capture (2016)	[71]
18	Various biomass	Carbonization, composites	Carbons and composites	Li-ion battery anodes (2017)	[72]
19	Auricularia (mushroom)	Balanced meso/micropore synthesis	Hierarchical graphene–carbon hybrids	Flexible supercapacitors (2015)	[73]
20	Lignocellulosic biomass	Fe-catalyzed graphitization	Nanostructured graphitic carbon	Electrodes, filtration (2014)	[74]
21	Sugarcane bagasse, corn residues, tire chips, post-consumer PP/PET	Pyrolysis and partial oxidation	Carbon nanomaterials	Bulk production (2012)	[75]
22	Food waste	Carbonization, activation	Porous carbons, carbon dots	Environmental remediation, sensors (2024)	[28]
23	Lignocellulose (cellulose, lignin)	Carbonization, activation	Carbon dots, nanofibers	Bioimaging, sensors, LEDs (2023)	[76]
24	Lignocellulosic biomass	Green synthesis, carbonization	Carbon dots, nanofibers	Water treatment, biomedical diagnostics (2022)	[77]

**Table 2:** Properties and scale-up potential of carbon-based nanomaterials produced by green synthesis.

		Reaction Time	Type	CNM Characteristics	Treatments Additional	Scale-Up	References
Pyrolysis		Hours	CDs Graphene-based materials	High purity and adjustable porosity.	-	Requires high temperature.	[140]
Hydrothermal/solvothermal		Hours	CDs, GDs, Nanospheres, Porous carbons	Precursor-dependent.	Purification processes	Depends on carbon sources and solvent concentrations. Requires large amounts of solvent.	[140]
			CDs Doped-Materials with N, S and/or B	Uniform size. Flexible control over reduction degree. Heteroatom doping.	Filtration and drying	Scalable. High yield.	[135]
Hydrothermal carbonization		24 h to 180 °C	Doped-CD with N	One-step conversion of biomass to carbon-rich materials. Adjustable morphology.	Purification processes	Allows industrial waste processing.	[140]
Ultrasonication		Minutes–hours	CDs Graphene CNTs Nanodiamonds	High quality. Adjustable size and morphology. High photostability. Heteroatom doping.	Calcination or filtration	Limited scalability depending on precursor.	[142,156]
Microwave		≥30 min	CD	Uniform size.	Purified trough centrifugation, filtration and drying	Scalable. High yield.	[135,140]
		Seconds	CNTs Palladium-graphene Oxide-based CNTs	Flexible control over reduction degree. Heteroatom doping.	-	Flexible control over reduction degree.	[157]
Plasma-assisted Synthesis		Minutes	GQD Doped–CQD with N. CNTs	High purity.	Simple purification processes	With scalability potential.	[135]
		15 min	Nanospheres	Small size (less than 50 nm). Flexible control over reduction degree. Doping regulation.	Simple purification processes	High conversion efficiency.	[158]
Hybrid/combined methods	Microwave- assisted Pyrolysis	≥11 min	Water-soluble CQD	High photoluminescence quantum yield. Reduced size.	-	Scalable. Feasible for mass production.	[154]
	Microwave- assisted hydrothermal.	12 h	NIR–CD with o-PDA	Fluorescence emission tuning.	Filtration	Scalable kilogram-scale synthesis with extremely low cost (0.1 dollar/g).	[159]
		Minutes	CDs	Accurate control over size, morphology and surface properties. High photoluminescence. Doping with S and N. Ultraviolet-absorption properties.	-	Reduced production time and more uniform products.	[142]
			CDs	Particle size depends on biomass source and process temperature.	Freeze-drying	Biomass source dependent.	[145]
	Solvothermal ultrasound-assisted	-	Sulfur-doped rose-like carbon nitride (RCN)	Adjustable morphology and doping with sulfur.	Calcination	Scalable.	[160]
	Ultrasound-assisted hydrothermal.		CDs	High quality. Control over size and morphology. Enhanced optical properties.	-	Efficient and rapid with scalability potential.	[142]

**Table 3:** Comparative overview of synthesis and activation strategies for biomass-derived carbon nanomaterials and their resulting physicochemical properties.

Synthesis Method	Typical Temperature Range	Dominant Pore Type/Morphology	Surface Functional Groups (General Trend)	Main Advantages	Main Limitations	References
Pyrolysis (O_2_-free)	≈300–800 °C	Hard, amorphous to graphitic carbons, micro/mesoporosity, CNTs/nanofibers at high T with catalysts.	Low T: O-rich surfaces. high T: oxygen loss and graphitic ordering.	Scalable and versatile, wide range of carbon nanostructures.	High energy demand, limited porosity control without activation, loss of surface polarity at high T	[128,129,130,131]
Carbonization (biomass/MOFs)	≈500–900 °C (biomass); up to ~1400 °C (highly graphitized/MOF-derived)	Tunable micro-/mesoporosity, possible closed pores.	Decreasing O content, heteroatom or metal doping from precursors.	Good control of texture, suitable for doped and MOF-derived carbons.	Often requires activation, energy intensive.	[27,128,129,130,132,133,134]
Hydrothermal and solvothermal	120–250 °C; up to ~350 °C	Spheres, dots, sheets, moderate porosity, mostly amorphous.	Abundant O/N functional groups.	Low-temperature, green processes, good morphology control.	Low surface area and conductivity without post-treatment.	[135,136,137,138]
Microwave-assisted synthesis	Typically 120–300 °C	Carbon dots, nanoparticles, porous carbons with uniform size.	Highly functionalized (N, O, S), tunable surface chemistry.	Fast, homogeneous heating, high yields, short processing times.	Limited industrial scale: MW field control required	[135,139,140,141]
Ultrasonication	Ambient to mildly elevated	Exfoliation and fragmentation of existing carbons, defect generation.	Mild oxygen functionalization, improved wettability.	Enhanced dispersion, effective post-treatment.	No intrinsic carbon synthesis, risk of structural damage.	[120,135,140,142,144,145,147]
Plasma-assisted synthesis/functionalization	Near ambient to <500 °C (macroscopic)	Surface activation, nanoparticle redistribution, ultrathin doped carbon shells.	High density of reactive sites, efficient heteroatom doping.	Fast, dry, single-step, precise NP control, avoids harsh chemicals.	Limited penetration depth, scale-up challenges, specialized equipment.	[149,150,151,152,153]

**Table 4:** Dominant structure–property–performance relationships governing pollutant removal by biomass-derived carbon nanomaterials.

Class of Pollutant	Dominant Material Property	Primary Removal Mechanism	Typical Synthesis/Activation Route
Heavy metals (Pb^2+^, Cr^6+^, Cd^2+^)	Abundant surface –COOH/–OH groups and N-dopants	Chelation/complexation and ion exchange (surface complexation, redox for Cr^6+^)	Biochar pyrolysis followed by chemical activation (e.g., KOH/steam); heteroatom doping (NH_3_, urea)
Dyes (cationic/anionic)	Charged surface functional groups; aromatic π-surface	Electrostatic attraction (to opposite charges), π–π stacking, H-bonding, pore filling	Acid or alkali activation (H_3_PO_4_, KOH) to create micro/mesopores; tuning pH
Antibiotics (e.g., tetracycline)	High surface area, π-conjugated domains, polar moieties	π–π interactions, hydrogen bonding, electrostatic interactions (pH-dependent)	Pyrolysis-derived biochar, often with N-doping or alkaline modification
Aromatic organics (phenols, EDCs)	High aromaticity (graphitic domains) and hydrophobic microporosity	π–π donor–acceptor interactions, hydrophobic partitioning	High-T pyrolysis (to maximize graphitization) and activation (CO_2_, KOH) for microporosity

## Data Availability

Data sharing is not applicable.
